# Interactions Between Gut Microbiota and Acute Childhood Leukemia

**DOI:** 10.3389/fmicb.2019.01300

**Published:** 2019-06-19

**Authors:** Yuxi Wen, Runming Jin, Hongbo Chen

**Affiliations:** Department of Pediatrics, Union Hospital, Tongji Medical College, Huazhong University of Science and Technology, Wuhan, China

**Keywords:** gut microbiota, acute childhood leukemia, immune system, long-term health problem, diet construction

## Abstract

Childhood leukemia, the commonest childhood cancer, mainly consists of acute lymphoblastic leukemia (ALL) and acute myeloid leukemia (AML). Though great progresses have been made in the survival rates of childhood leukemia, the long-term health problems of long-term childhood leukemia survivors remain remarkable. In addition, the deep links between risk factors and childhood leukemia need to be elucidated. What can be done to improve the prevention and the prognosis of childhood leukemia is an essential issue. Gut microbiota, referred to as one of the largest symbiotic microorganisms that is accommodated in the gastrointestinal tract of human or animals, is found to be involved in the progression of various diseases. It is reported that microbiota may keep people in good health by participating in metabolism processes and regulating the immune system. Studies have also explored the potential relationships between gut microbiota and childhood leukemia. This review is meant to illustrate the roles of gut microbiota in the onset of acute childhood leukemia, as well as in the progress and prognosis of leukemia and how the treatments for leukemia affect gut microbiota. Besides, this review is focused on the possibility of building or rebuilding a healthy gut microbiota by adjusting the diet construction so as to help clinicians deal with childhood leukemia.

## Introduction

Leukemia, the commonest childhood malignancy, mainly consists of acute lymphoblastic leukemia (ALL) and acute myeloid leukemia (AML) (Steliarova-Foucher et al., 2017). Over the past decades, tremendous progresses have been made in the cure of childhood leukemia. The mortality rates of childhood cancers in the United States have decreased by more than 50% from 1975 to 2010, and the 5-year survival rate for ALL children <15 years old has increased to 91%, while the survival rate for AML children has increased to 68% (Smith et al., 2014). Genetic background, birth weight, birth order (Crump et al., 2015a,b; Paltiel et al., 2019), caesarean delivery (Marcotte et al., 2016), breastfeeding (Amitay and Keinan-Boker, 2015), low dose of ionizing radiation (Little et al., 2018), and some other exposures are reported to influence the incidence of childhood leukemia. However, the deep links between these factors and acute childhood leukemia lack exploration, and the exact mechanisms for acute childhood leukemia are still not clear.

Gut microbiota is recently recognized as a factor that could be important in regulating the progress of diseases (including gut diseases, diabetes, and others). Microbiota, microorganisms that accommodate at various sites of the human or animal body, develops during the first few years of life and then lives in symbiosis with humans all their life (Arrieta et al., 2014; Hollister et al., 2015; Cheng et al., 2016). Gut microbiota is considered to be one of the largest and most complex ecosystem that is coevolved with the gastrointestinal tract. Since several factors such as genetic, environmental, and lifestyle can influence microbial constitutions (Little et al., 2018; Rothschild et al., 2018), these factors along with gut microbiota should be evaluated as integrated for cancer progress. The aim of this review was to figure out current understandings on interactions between gut microbiota and acute childhood leukemia and make out what can be done in future studies for the management of childhood leukemia.

## Gut Microbiota Changes Rapidly During Childhood and Should Be Taken Into Account in the Future Study Design

With the development of gene sequencing methods, studies are able to be carried out to identify the constitution and diversity of microbiota as well as the crucial roles of microbiota in maintaining body health and regulating the progress of diseases. Several body sites (including the gastrointestinal tract, oral, skin, etc.) have been identified to harbor microbiota, among which the gastrointestinal tract is the largest and most complex one, which harbors approximately 100 trillion microorganisms (mainly composed of bacteria) in the human body (Bull and Plummer, 2014; Valdes et al., 2018). The roles of gut microbiota in maintaining body health have been explored (Beaumont et al., 2016; Falony et al., 2016; De Palma et al., 2017; Little et al., 2018), and gut microbiota is found to be vital for humans, probably by participating in metabolism, regulating the movement and development of the intestinal tract, promoting the development of the brain, as well as regulating the immune system (Zhang et al., 2015b; Levy et al., 2017; Chen et al., 2018; Rothschild et al., 2018; Valdes et al., 2018). With so many diseases discovered to be associated with gut microbiota, researchers are passionate about figuring out the roles of gut microbiota in various fields so as to find out something new for the clinical diagnosis and management of diseases.

**Figure 1:**
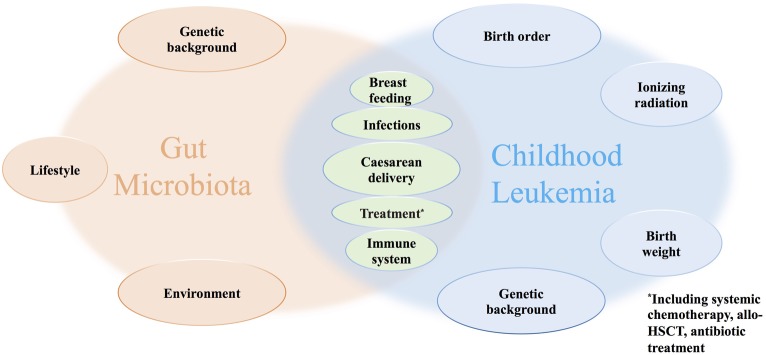
Factors that may disturb the composition and diversity of gut microbiota and influence the progress of childhood leukemia.

Ever since birth, gut microbiota has interacted with the host’s conditions and shaped by numerous factors, such as genetic, diet construction, drugs, and others (Figure 1) (Little et al., 2018; Rothschild et al., 2018). Though the gut microbiome is divergent from people to people, the constitution and function of an individual’s gut microbiota remain relatively stable, which is quite important for maintaining health (Lozupone et al., 2012; Moya and Ferrer, 2016). Studies believed that gut microbiota is established at the first few years of life and keeps developing during the childhood until adulthood (Arrieta et al., 2014; Hollister et al., 2015; Cheng et al., 2016). A cohort study carried out among healthy preadolescent children with ages from 7 to 12 years found that the diversity of gut microbiota was at a similar level in healthy children and adults, while the composition and function of the microbiome differed. It mainly consists of *Bifidobacterium* spp. and *Faecalibacterium* spp. for children, while it mainly consists of *Bacteroides* spp. for adults. As for functional differences, most of the children’s microbiota are found to be able to promote development, while those of adults mostly participate in inflammation, obesity, etc. (Hollister et al., 2015). Another study that included children 1–4 years old drew similar results (Cheng et al., 2016). The gut microbiota community is found to change rapidly during the first few years and stay stable in the following years of adulthood until the decline of stability and function of the microbiota community for elders (Lan et al., 2013; Arrieta et al., 2014; Hollister et al., 2015; Cheng et al., 2016; An et al., 2018).

Differences in composition of the gut microbiota between healthy children and healthy adults, as well as the rapid change in childhood gut microbiota composition, stressed the importance of figuring out the specific vital leukemia-causing microorganisms based on a baseline childhood microbiota diversity and constitution. Arrieta et al. (2014) believed that the development of gut microbiota can be divided into six stages (at birth, 1 month, 6 month, 12 months, 3 years, and more than 3 years old) since the composition and diversity differed for every stage. For future studies on gut microbiota and childhood leukemia, the altering of gut microbiota along with aging should be taken into account. A recent study of gut microbiota, which involved 7,009 individuals from 14 districts in Guangdong province, showed that the locations of the hosts were associated with the variations of microbiota (He et al., 2018). Though there could be some confounding for this result, it reminds us that regional diversity is another factor which should be taken into account during the designing of future studies about microbiota.

## Studies About the Possible Relationships Between Gut Microbiota and Leukemia

Since the incidence of ALL for children is much higher than that of AML (Steliarova-Foucher et al., 2017), studies for childhood AML are quite rare, and for better understanding, some studies about adult AML are included here. Nearly all children with leukemia are treated with systemic chemotherapy, and some may even receive allogeneic hematopoietic stem cell transplantation (allo-HSCT). Drugs used in chemotherapeutic and antibiotic treatments are known to disturb the host gut microbiota (Holler et al., 2014; Keeney et al., 2014) and, as a result, damage the mucosal protection and immunologic balance, and then contribute to the inflammation of the intestine (Holler et al., 2014).

## Which Specific Types of Infection Are the Protective or Detrimental Factors for the Occurrence of Childhood Leukemia

According to a landscape study, childhood cancers are frequently driven by a single disease-specific mutation, which is quite different from the mechanisms for adulthood cancers (Bandopadhayay and Meyerson, 2018). The most common alterations for pediatric leukemia are CDKN2A, IKZF1, ETV6, and RUNX1, which mainly participate in the regulation of cell cycle and transcription (Ma et al., 2018). Mutations in PAX1 (transcription) and NOTCH1 (notch) were only found in ALL, while mutations in CBFB (transcription) were only found in AML (Ma et al., 2018). For some subtypes of pediatric leukemia, the “two genetic hits” hypothesis proposes that a secondary genetic change is indispensable for the arisen on the basis of a fusion gene or hyperdiploidy ever since in the utero (Knudson, 2001; Greaves, 2018). It can be best verified by the decreasing concordance rate with aging for monochorionic twins. Although monochorionic twins are considered to share the same initial genetic change (with equal preleukemic stem cells), a secondary genetic change is believed to be the cause for the condition that only one of the twins develops ALL in children (Cazzaniga et al., 2011; Bateman et al., 2015) or AML in adults (Jaiswal et al., 2014; Shlush et al., 2014).

More than 20 possible exposures, such as prenatal factors (Marcotte et al., 2014; Crump et al., 2015a,b), caesarean delivery (Marcotte et al., 2016), breastfeeding (Amitay and Keinan-Boker, 2015), low dose of ionizing radiation (Little et al., 2018), as well as infections, have been reported to be related with the occurrence of acute childhood leukemia. The “delayed infection hypothesis” believed that earlier exposures to microbiome are protective factors for childhood ALL, while the later infections without earlier exposures may contribute to the vital secondary variation that causes leukemia (Greaves, 2018). Studies of factors that are associated with exposures to infections (such as birth order, timing of birth, and caesarean delivery by which children were not exposed to the microbes in the maternal vaginal) can support this hypothesis to a certain degree (Marcotte et al., 2016). The reduction of exposures to early common infections as well as factors that relate with microbiota colonization (such as breastfeeding, vaginal delivery) were considered to increase the risk of childhood leukemia (Ajrouche et al., 2015), while some other studies claimed that medically diagnosed infections in infancy or before diagnose of ALL or AML indicate increased risks for childhood leukemia (Chang et al., 2012; Rudant et al., 2015). As for the development of *de novo* adult AML, only gastrointestinal infections were considered to be risk factors (Ostgard et al., 2018).

The different conclusions of the different studies may come from the sample selection. By figuring out the earlier stage of infection and analyzing the relationship between acute childhood leukemia and earlier or later stage infections separately, conclusions might be more reliable. Studies stressed the fact that the immune system and microbial infectious exposures influence each other both *in utero* and in infancy (Olszak et al., 2012; Lim et al., 2015; Laforest-Lapointe and Arrieta, 2017; Torow and Hornef, 2017; Haas, 2018). However, how to define early-stage infections as well as how to prove the existence of it are still unsolved. Signe found that newborns who develop B-ALL later are characterized by abnormal concentrations of several inflammatory markers (Soegaard et al., 2018). The abnormal concentration of inflammatory markers represents an abnormal immune function at birth and reminds us that the immune function might play important roles in the development of acute childhood leukemia. The early childhood exposures to infection were associated with the proliferation and expansion of B or T cell clones (Olszak et al., 2012), and early common infections before the maturity of CD4 T cells are likely to adjust the constitution of symbiotic gut microbiota and contribute to an immune tolerance state toward some antigens with the aid of regulatory T cells and sIgA from mothers (Torow et al., 2015). The excessive reaction toward later-stage infection is likely to be the trigger of acute childhood leukemia. However, recent retrospective studies which rely on maternal recall are limited to figure out the existence of earlier-stage infections. Further researches are in great need to identify whether the earlier exposure to specific microorganisms reduces the incidence of childhood ALL, as well as childhood AML, by regulating the gut microbiota and thus contributing to the building of a healthy immune system.

## Interactions Between Gut Microbiota and Treatments for Childhood Leukemia

Myelosuppression and immunosuppression are common conditions for children with leukemia during anticancer therapeutics. Infections (mostly bloodstream infections) that are followed by myelosuppression and immunosuppression play important roles in the morbidity and mortality for childhood leukemia. The disturbance of the gut microbiota during chemotherapy procedures and allo-HSCT in children with leukemia has been explored. Changes of stool microbiota were examined in a large cohort study of children with ALL to reflect the changes of gut microbiota. The diversity of fecal microbiota reduced remarkably after induction and reinduction chemotherapy (Hakim et al., 2018). Hakim et al. (2018) believed that the presentation of *Proteobacteria* including *Enterobacteriaceae* and *Pseudomonas* species, and other bacteria in the gut microbiome before or during chemotherapy could be used for predicting subsequent outcomes such as diarrhea, bloodstream infections, or febrile neutropenia for childhood leukemia. Similar conclusions have also drawn in some adult allo-HSCT, AML, and non-Hodgkin lymphoma (Montassier et al., 2015; Taur et al., 2015; Galloway-Pena et al., 2016). In the large cohort study, though the diversity of gut microbiota could recover to the initial level, the composition was differed. The composition of the microbiota in children instead of the diversity in adults was identified to be independently predictive of infections caused by immunosuppression during chemotherapy (Hakim et al., 2018). Another study believed that the diversity and composition of gut microbiota before treatment can be applied to predict chemotherapy-related bloodstream infections (Montassier et al., 2016). However, a study proposes that stool microbiota is quite different from the microbiota that is detected from intestinal mucosa (Zmora et al., 2018). So, more studies are needed to confirm this opinion and to identify the representativeness of stool microbiota for gut microbiota.

Several studies illustrated that the disturbance of microbiome caused by antibiotics is not always temporarily, but in some cases continues (Hernandez et al., 2013; Perez-Cobas et al., 2013; Vangay et al., 2015). Antibiotic-induced shifts can increase the susceptibility toward *Clostridioides difficile* infection (Hernandez et al., 2013). Methotrexate (MTX) is widely used in the treatment for childhood leukemia. Studies showed that the gastrointestinal toxic induced by MTX is vital for patient management (Paci et al., 2014). A mice study showed that the disturbance of the gut microbiota for wild-type mice resulted in a tendency of suffering from MTX-induced mucosal injuries (Frank et al., 2015). A review claims that the microbiota interacts with anticancer drugs mainly in three aspects: improving the drug efficacy, reducing the anticancer effect, and increasing or reducing the toxicity (Panebianco et al., 2018).

The interactions between gut microbiota and therapeutic processes for childhood leukemia can be identified from divergent aspects: (1) whether the diversity and composition of gut microbiota can influence the efficacy or toxicity of drugs used during the therapeutic processes and how; (2) whether the treatments (chemotherapy or allo-HSCT) disturb the gut microbiota and how; (3) whether the gut microbiota can be used for predicting therapy-related complications (such as infections and diarrhea); and (4) whether it is possible for clinicians to deal with long-term health problems or therapy-related complications by regulating the gut microbiota.

## What Can Diet Regulation Do Both for Gut Microbiota and Acute Childhood Leukemia

Since the balance of gut microbiota is rather important in childhood leukemia, efforts made to regulate or adjust the gut microbiota to a healthy state are in great need. Compared with administration of a multistrain probiotic preparation, the postantibiotic gut microbiota both in human and murine was rebuilt to the initial state more quickly by autologous fecal microbiome transplantation (Suez et al., 2018), similarly to three other researches (Yan et al., 2016; Bidu et al., 2018; Smillie et al., 2018; Suez et al., 2018). Besides, symbiosis between host and bacterium was believed to be dominantly driven by the bacterium’s adaptation to the host’s diet in a *Drosophila* model (Martino et al., 2018). Another study believed that melatonin (which is sufficient in several foods) supplementation can increase the diversity and regulate the composition of gut microbiota in mice (Ren et al., 2018). A meta-analysis that involved 18 studies believed that by breastfeeding for at least 6 months, the risks of childhood leukemia were reduced significantly compared with no or shorter-time breastfeeding (Amitay et al., 2016). There are sufficient prebiotic and antibodies for specific pathogens (which each infant’s mother is exposed to) and much more natural killer cells in breast milk, which are essential for building a healthy microbiota in the gastrointestinal tract (Benno et al., 1984; Bode and Jantscher-Krenn, 2012; Brown, 2013). Thus, we can assume that the different outcomes of divergent diet constructions might come from the altered gut microbiota which evolved with the human immune system, especially for infants and children.

Long-term childhood leukemia survivors are faced with many long-term health problems such as obesity, cardiopulmonary toxicity, secondary malignancy, late neurotoxic effect, and others (Essig et al., 2014; Zhang et al., 2014; Cheung and Krull, 2015; Withycombe et al., 2015; Duncan et al., 2018). The unhealthy dietary behaviors (such as high intake of fat, sodium, sweets, and low intake of fruit, vegetables, and whole grains) (Tylavsky et al., 2010; Badr et al., 2011; Fuemmeler et al., 2013; Zhang et al., 2015a; Duncan et al., 2018) of childhood cancer survivors and the usage of antibiotics are known to increase weight by regulating the composition of the gut microbiota (Blaser, 2016). Obesity was identified to be a risk for childhood leukemia, while fasting was found to reduce the incidence and even reverse the progression of ALL in mouse models (Orgel et al., 2016; Lu et al., 2017). Hopefully, the incidence and progression of leukemia are likely to be stopped or reversed by simply fasting; prolonging the duration of breastfeeding; adjusting to a low intake of fat, sodium, and sweets; and a high intake of fruit, vegetables, and whole grains. Besides, whether the supplement of melatonin or probiotics, as well as fecal microbiome transplantation, can help keep a healthy gut microbiota in children with leukemia remains to be explored.

## Conclusions

Recent studies have explored the possible relationships between gut microbiota and acute childhood leukemia. The “delayed infection hypothesis” highlights a favorable role of early infection in preventing the onset of childhood leukemia. This is somehow consistent with the idea that an oversanitized condition may lead to some noninfectious and immunological diseases like asthma, obesity, and diabetes. It is also found that gut microbiota develops with ageing. However, which specific microorganisms help push the onset and progression of childhood leukemia is still unclear. Besides, diet regulation such as fasting and breastfeeding may lower the incidence of childhood leukemia and reverse the progression by adjusting the gut microbiota. All of the above implies that acute childhood leukemia may be somewhat preventable by changing the lifestyle. In turn, antileukemia therapies can disturb the gut microbiota and bring short- and long-term health problems. This may be addressed in the future by gut microbiota transplantation and probiotic supplements, which demands some prospective studies in leukemia patients first.

There is also an urgent need to figure out the specific microorganisms for the onset and progression of childhood leukemia and dig out the latent mechanisms for the links between gut microbiota and childhood leukemia. By doing so, more targeted therapy (such as specific probiotic supplements, specific microbiome transplantation, as well as diet regulation that benefits specific microorganisms) instead of nontargeted probiotic supplements or others for reducing the incidence of leukemia and eliminating antileukemia treatment-related long-term health problems could be put forward. As for study design, both regions and ages should be taken into account to generate a confident baseline childhood microbiome diversity and constitution. In addition, the representativeness of stool microbiota for gut microbiota is in doubt and needs further confirmation.

## Author Contributions

All authors listed have made a substantial, direct and intellectual contribution to the work, and approved it for publication.

### Conflict of Interest Statement

The authors declare that the research was conducted in the absence of any commercial or financial relationships that could be construed as a potential conflict of interest.
